# The Mechanism of Creep during Crack Propagation of a Superalloy under Fatigue–Creep–Environment Interactions

**DOI:** 10.3390/ma13194418

**Published:** 2020-10-04

**Authors:** Minqing Wang, Jinhui Du, Qun Deng

**Affiliations:** Beijing Key Laboratory of Advanced High Temperature Materials, Central Iron and Steel Research Institute, Beijing 100081, China; dujinhui@cisri-gaona.com.cn (J.D.); dengqun@cisri-gaona.com.cn (Q.D.)

**Keywords:** dwell-fatigue-crack growth rate, creep, oxidation-induced crack closure, grain boundary

## Abstract

In this study, we examine the mechanism of fatigue-crack propagation in 718Plus superalloy at 704 °C under fatigue–creep–environment interactions, in this case, a new turbine disc material used in aero-engines at high temperatures. The effect of creep on the fatigue-crack propagation of the superalloy at high temperature was also researched. There was an unusual inhibitory effect on the propagation of fatigue cracks in 718Plus alloy, in which the propagation rate of fatigue cracks decreased with the increase of creep time through exploration of dwell-fatigue-crack growth (DFCG) test with different creep times. In particular, under lower stress intensity factor range (Δ*K*) conditions, the fatigue-crack growth rate with a 90 s hold-time was one order of magnitude lower than that with a 5 s hold-time. Conversely, the gap between the two DFCGs gradually decreased with the increase of Δ*K* and the creep effect became less apparent. The mechanism of crack propagation in 718Plus alloy under two creep conditions was investigated from a viewpoint of the microstructure, oxidation rate at high temperature and crack path morphology under different conditions.

## 1. Introduction

The nickel-based superalloy 718Plus alloy combines the high temperature resistance of Waspaloy and the workability of Inconel 718 alloy. Hence, this alloy attracts much attention from researchers due to its potentiality in the manufacture of advanced aerospace engines—especially for turbine discs, in which it must withstand complex interactions of fatigue, creep and oxidation at high temperatures under alternate loading and high temperature corrosion environments [1,2,3]. Microstructure strongly determines the final mechanical properties of turbine discs produced by forging. However, most investigations of 718Plus superalloy in public have focused on the metallurgical process [4,5,6], phase precipitation and transformation during heat treatment [7,8,9], crack propagation and failure behaviors [10,11,12,13], welding [14,15,16,17], etc. Kennedy et al. [1] measured fatigue-crack growth rates of Inconel 718, Waspaloy and 718Plus alloys, where the tests were performed at 650 °C and 704 °C with a zero and a 100 s hold-time. The results showed that crack-growth rate for 718Plus alloy was lower than those of either Inconel 718 or Waspaloy superalloys without hold-time. Moreover, 718Plus alloy performed better than 718 alloy but not as well as Waspaloy alloy with a 100 s hold-time. Liu et al. [18] found that the crack path of 718Plus alloy was predominantly intergranular, with a small transgranular cleavage fraction under 3 + 100 s hold-time conditions at 650 °C and 704 °C. The fracture surface was rough and covered by a large amount of oxide products. Timothy et al. [19] investigated the effects of hot corrosion pits on low-cycle fatigue life and failure modes of the disk superalloy ME3 and found that hot corrosion pitting can occur for disk superalloys with salt deposits and exposures near 704 °C. These pits can significantly reduce low-cycle fatigue life at low and high temperatures. Shot peening can induce near-surface compressive residual stresses which appear able in some cases to mitigate the effects of corrosion pits on fatigue life. However, the mechanism of creep on the fatigue-crack propagation of 718Plus alloy remains controversial to this day. Many researchers got different conclusions based on their observations and proposed varied mechanism theories of creep on the fatigue-crack propagation in superalloys [18,19,20,21]. In general, the oxide-induced embrittlement increased at the grain boundaries, leading to the aggravation of creep damage, thus, fatigue-crack propagation accelerated. Moreover, the effect of creep and oxidation increased significantly with the increase of hold-time. Therefore, the propagation rate of fatigue crack in the alloy tends to increase as the hold-time increases [22]. However, the literature [20] reported that creep suppressed the propagation of fatigue cracks within a low stress intensity factor range (Δ*K*) at ~760 °C, which occurred due to the creep-induced stress relaxation and stress concentration at the crack tip. Hence, the effect of creep is complicated and depends on different alloys, temperature and environment, etc.

The dwell crack-growth rate of turbine disc materials and predictive models for operating life are essential in the field of superalloys. The operating condition is a complicated process involving several factors such as fatigue, creep and environment. Therefore, the crack-growth rate of turbine disc materials under fatigue–creep–environment interaction is difficult to predict and the research regarding the mechanism and the models is still in the preliminary stage. There is a lack of relevant experimental data so far. Thus, it is essential to investigate the mechanism of crack-growth rate under the fatigue–creep–environment interaction for the long-term reliability of 718Plus components and the safe operation of an aero engine. Based on the above background, this study simulated and predicted the crack propagation during the process of start-run-stop of the aero-engine. The effect of creep on superalloys at high temperature was investigated. Moreover, the fatigue-crack propagation behavior and fracture morphologies with different hold-times were analyzed. Finally, the mechanism involved in the effect of creep on crack propagation in 718Plus alloys was discussed.

## 2. Experiment Materials and Procedure

### 2.1. Materials

718Plus alloy using for the experiment was prepared by vacuum induction melting (VIM-50) and vacuum arc remelting (VAR, K-DHM-600, Jinzhou Tian Yu Electric Stove Co., LTD, Jinzhou, China). Then, the 50 kg ingot was homogenized by heating treatment in furnace and rolled into bars with a diameter of 40 mm and then were cut into test samples. The chemical composition of the experimental alloy is listed in Table 1. All the samples were subjected to solution heat treatment at 954 °C for 1 h, air cooled and then aged at 788 °C for 8 h, furnace cooled at 56 °C/h to 704 °C, held for another 8 h and finally air cooled.

The microstructure of the alloy samples was analyzed by optical microscopy (OM, XSP-3CA, Shanghai Optical Instrument Factory, Shanghai, China), scanning electron microscopy (SEM, JEOL JSM-6480LV, Tokyo, Japan) and field-emission SEM (FESEM, ZEISS SUPRA 55, Oberkochen, Germany).

### 2.2. Test of the Fatigue-Crack Propagation

**Figure 1:**
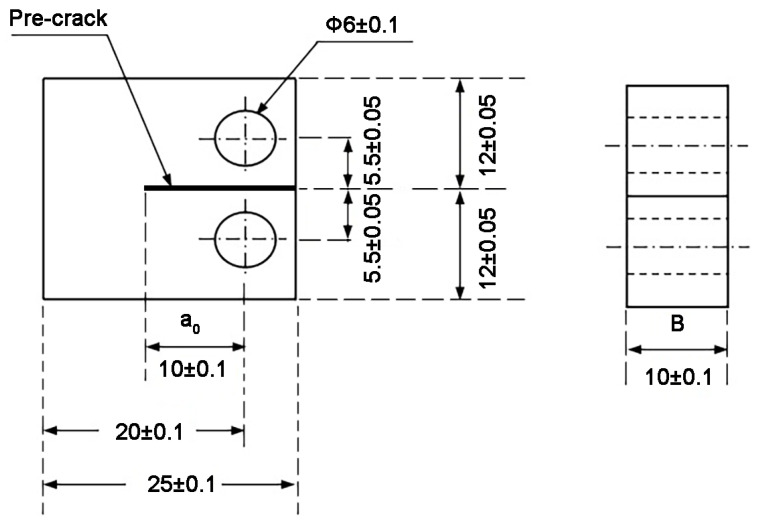
Compact tension specimen configuration used for crack growth tests, dimensions in mm.

Figure 1 shows the specimen used for crack growth test, which was according to the standard compact tension (CT) specimen configuration. The crack plane was parallel to hot working direction and crack growth was along the radial direction. The specimens were pre-cracked using machining methods such as wire cutting and milling at ambient temperature, which were then heated to a higher temperature for the fatigue-crack growth test. The dimensions of CT specimen are shown in Figure 1.

**Figure 2:**
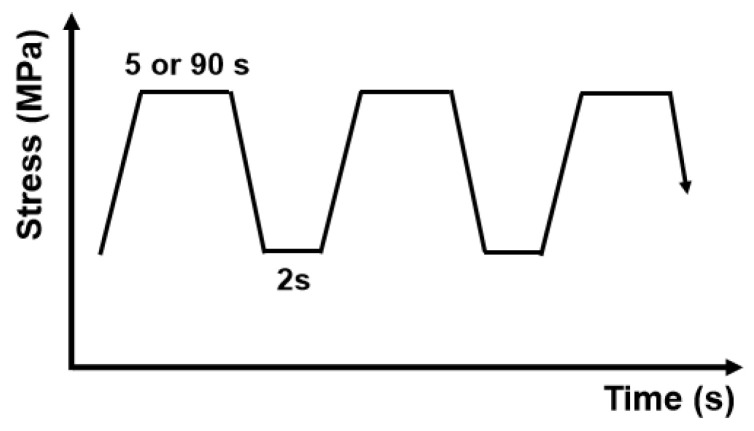
Trapezium waveform used in fatigue–creep–environment interaction-loading conditions: 15 s (Loading time)–5 s (Holding time)–15 s (Unloading time)–2 s (Intermittence time) and 15 s (Loading time)–90 s (Holding time)–15 s (Unloading)–2 s (Intermittence).

The tests were carried out at 704 °C under loading control process, which was a trapezoidal wave, as shown in Figure 2, using two loading modes represented of 15 s-5 s-15 s-2 s and 15 s-90 s-15 s-2 s, respectively. The stress intensity ratio (*R* = *K*_min_/*K*_max_) was set to 0.05. The crack zone of CT sample was mechanically polished and etched. Analysis of fracture morphology was conducted by SEM. The increment in the crack length during fatigue and sustained crack growth tests was monitored continuously by an Electro-hydraulic servo-controlled material testing machine (Instron 8501, Instron Limited, Hayward, UK). The machine could test fatigue-crack propagation rate of the material under vacuum, air or other gases, where the vacuum degree can reach up to 1 Pa. Three-point bend testing was conducted for the crack propagation rate measurement, and the length of crack was measured with the potential method [23,24].

The propagation paths of fatigue cracks in 718Plus alloy subjected to waveforms of 15 s-5 s-15 s-2 s and 15 s-90 s-15 s-2 s at 704 °C under air condition were observed by SEM. The waveform was unloaded after the cracks propagated to a certain extent, and the sample was observed via an electron microscope. The specimens were polished prior to testing. The type of oxide at crack tip in 718Plus alloy was measured using EDS in SEM.

## 3. Results

### 3.1. Microstructures and Mechanical Properties

**Figure 3:**
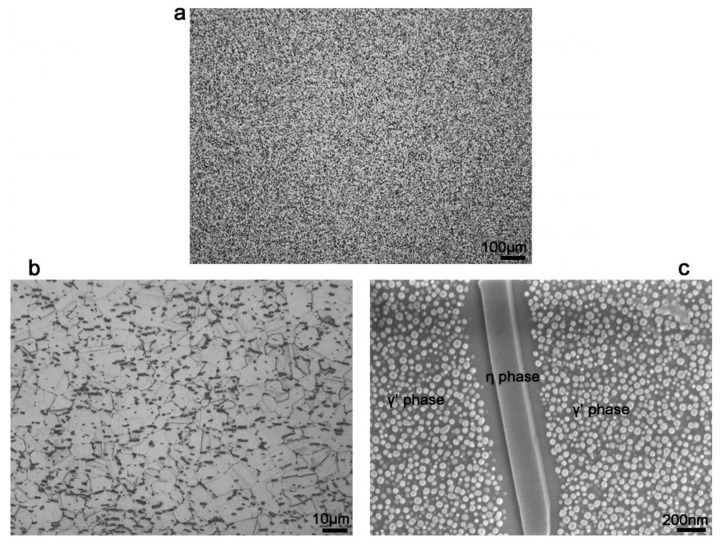
Microstructure of as-received dwell-fatigue-crack growth rate (DFCGR) specimen. (**a**,**b**) optical microscopy (OM) observation (grain size ~11 µm); (**c**) field-emission SEM (FESEM) observation.

Figure 3a,b shows OM observation of the microstructure of tested sample. The grain size of as-received samples was about 11 µm (according to ASTM 10 standard classification, i.e., *N*_AE_ = 2^G-1^, where *N*_AE_ is the number of grains contained in a square inch area within 100 times enlargement; G is the grain size degree [23]). The detailed microstructure of the samples was observed by FESEM, as shown in Figure 3c. It can be seen that a large amount of γ′ and η-Ni_3_Al_0.5_Nb_0.5_ phases precipitated after the solution and aging heat treatment. The precipitates showed a spherical shape, with size between 18 to 36 nm for the γ′ phase. It is evident that γ′ phase was depleted around η-Ni_3_Al_0.5_Nb_0.5_, which indicates that the η-Ni_3_Al_0.5_Nb_0.5_ precipitates grew at the expense of the γ′ strengthening phase. The precipitation of η-Ni_3_Al_0.5_Nb_0.5_ at the grain boundaries is known to restrict the slipping of grain boundaries and inhibit crack propagation, which would be beneficial for reducing the stress concentration and notch sensitivity [25]. The mechanical properties at high temperature (704 °C) of 718Plus superalloy are listed in Table 2. It can be found from table that the superalloy had high strength and plasticity at high temperature and was an excellent engineering material.

### 3.2. DFCGR Performances with Different Creep Times

**Figure 4:**
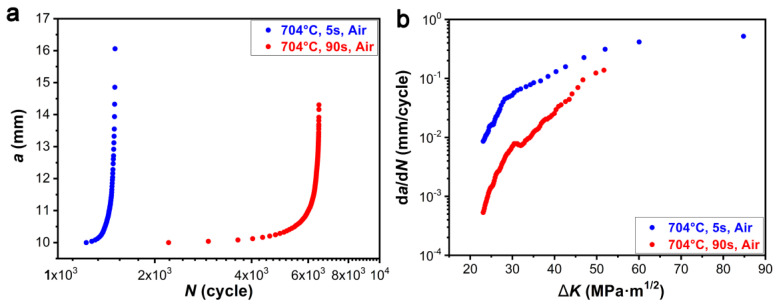
(**a**) *a*-*N* curves and (**b**) DFCGR curves with different hold-times (5 s, 90 s) of 718Plus alloy in air.

Dwell-fatigue-crack growth rate testing was performed at 704 °C and the initial Δ*K* value was 23 MPa·m^1/2^. The loading modes were trapezoidal waves with 15 s-90 s-15 s-2 s and 15 s-5 s-15 s-2 s, and the creep time per cycle were 90 s and 5 s, respectively. Figure 4 presents crack length-cycles number (*a-N*) curves and DFCG curves of 718Plus alloy at 704 °C with different creep times in air.

The influence of hold-time on the crack-growth rate is related to the testing environment (e.g., temperature, vacuum degree, etc.), loaded waveform and material type, etc. In general, the fatigue-crack growth rate of an alloy increases with the increase of hold-time, because of the effects of creep and oxidation. Consequently, the creep damage at the grain boundary is aggravated and the embrittlement phenomenon caused by oxidation occurs, that accelerate the expansion of cracks [26,27]. However, it was found that the acting time of creep had a significant effect on the fatigue-crack growth rate in 718Plus alloy (Figure 4), especially in the low stress intensity factor range (Δ*K*). Meanwhile, the fatigue-crack growth rate with a hold-time of 90 s was much lower than that with a hold-time of 5 s, i.e., the longer the creep hold-time was, the lower the fatigue-crack growth rate of 718Plus alloy was. In other words, creep inhibited the propagation of fatigue cracks in 718Plus alloy rather than promoting the crack propagation. In particular, the inhibition was more evident at lower Δ*K*. With the increase in Δ*K*, the difference between the fatigue-crack growth rates with 90 s and with 5 s hold-time became progressively lower. This indicated that the influence of creep was not significant when Δ*K* was in a higher level.

From Figure 4a, it could be obtained the fatigue-crack growth rate, the cycle number of crack initiation (*N*_q_), the starting cycle numbers of crack propagation (*N*_n_), the starting cycle numbers of crack final rupture (*N*_s_), as well as the cycle number of fracture (*N*_f_). In addition, the steady-state propagation proportion can be calculated as follows [28]:

*P* = (*N*_s_ − *N*_n_*)/N*_f_ (1)

where the proportion of steady-state propagation in the cycle number of fractures was also determined. A higher *p value* demonstrates a better ability to resist crack propagation. The initial proportion represents the proportion of initial process at the corresponding fracture cycle number, and can be calculated as following equation:

P* = *N*_n_*/N*_f_ (2)

A higher P* value indicates higher proportion of crack initiation at the fracture cycle number and better resistance against crack initiation. Table 3 presents the parameter values corresponding to the crack-growth rates of 718Plus alloy with two different hold-times (5 s, 90 s).

As observed from Table 3, the number of initiation cycles, transition cycles between initiation and propagation region and crack initiation ratio (P*) of 718Plus alloy with a hold-time of 90 s significantly increased compared to that with 5 s, indicating that 718Plus alloy had better resistance to initiation of fatigue cracks after 90 s hold time. In addition, the number of transition cycles between propagation and final rupture in 718Plus alloy with a hold-time of 90 s was higher and the steady-state crack propagation ratio (*P*) significantly increased, indicating of improved resistance to crack propagation. It was found that creep significantly inhibited the initiation and propagation of fatigue cracks in 718Plus alloys at 704 °C. In particular, the phenomenon was more evident at low Δ*K* value.

### 3.3. Typical Fatigue-Crack Fracture Morphology

**Figure 5:**
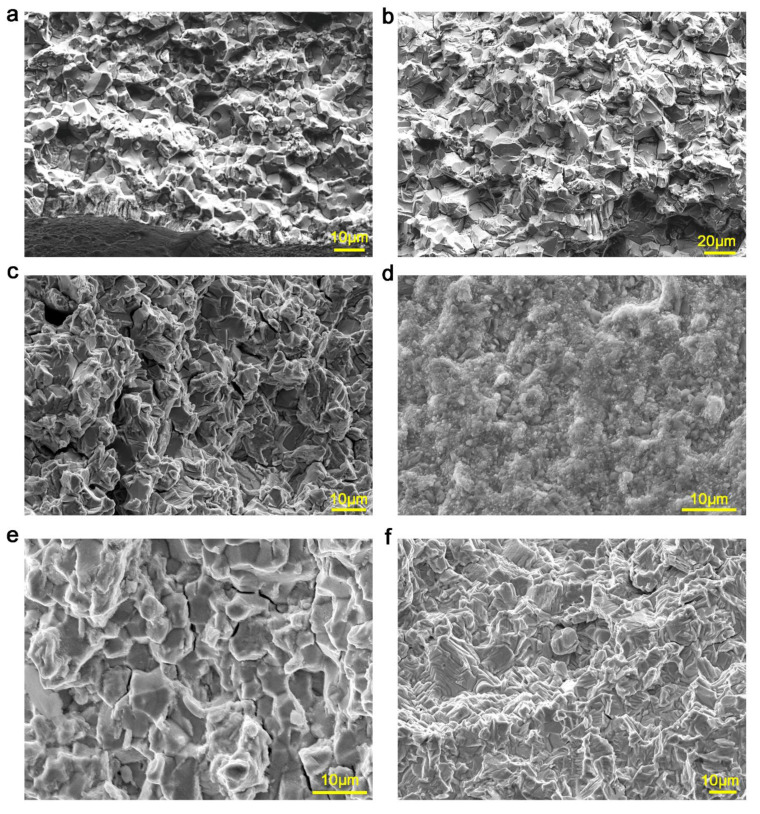
Fracture surface of fatigue cracks in 718Plus alloy with different hold-times. (**a**–**c**) 5 s hold-time; (**d**–**f**) 90 s hold-time; (**a**,**d**) initiation region; (**b**,**e**) propagation region; (**c**,**f**) rupture region of fatigue cracks, respectively.

Figure 5 presents the fracture morphology during fatigue-crack propagation in 718Plus alloy with the hold-time of 5 s (Figure 5a–c) and 90 s (Figure 5d–f) at 704 °C, respectively. It can be seen that both initiation regions of fatigue cracks with two hold-times exhibited obvious oxidation, where thick oxide layers were adhered to the fracture surfaces (Figure 5a,d). In particular, the oxidization degree of the specimen with a 90 s hold-time was more severe compared to that with 5 s hold-time. This was mainly due to the fact that the initiation region of the specimen with a 90 s hold-time was exposed to high temperature for a longer period. In the steady-state propagation region of fatigue cracks, both fracture propagation with two different hold-times displayed apparent intergranular morphologies, and a certain amount of oxides adhered to the surface (Figure 5b,e). Therefore, for the second region at the two fracture surfaces, fatigue cracks mainly propagated along the grain boundaries. Conversely, the final rupture position within the rupture zone with two hold-times exhibited a clear mixed grain structure and a surface almost without oxides (Figure 5c,f). According to the analysis for fracture morphology under different environments, fracture propagation of fatigue cracks in the air mainly exhibited intergranular morphology, whereas exhibited more transgranular morphology under low oxygen partial pressure environment. The results indicated that oxygen promoted the formation of intergranular cracks in 718Plus alloy.

### 3.4. Propagation Paths of Fatigue Cracks

**Figure 6:**
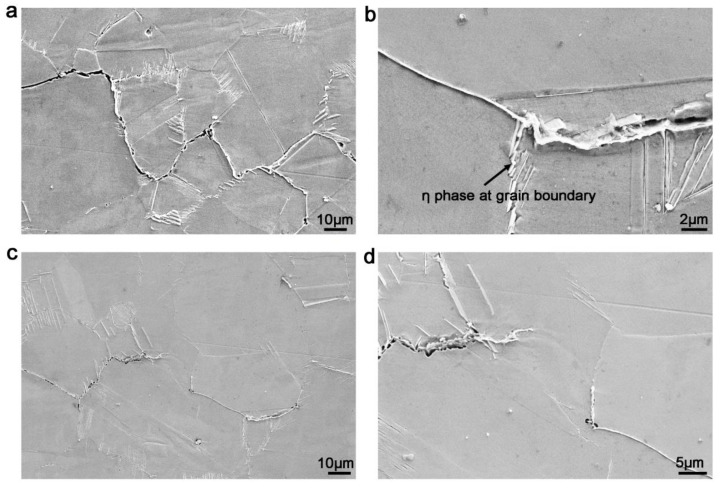
Crack propagation paths in 718Plus alloy with 5 s hold-time. (**a**) Propagation path; (**b**) precipitation at grain boundaries; (**c**) morphology at crack tip; (**d**) oxide strip at crack tip.

Figure 6 illustrates the propagation paths of fatigue cracks with the hold-time of 5 s. As can be seen, after 5 s hold-time at 704 °C, fatigue cracks in 718Plus alloy were initiated and propagated along the grain boundary, exhibiting a 45° angle along the stress. Subsequently, the cracks propagated almost perpendicular to the stress. Therefore, the crack microscopic propagation paths were of zigzag shape around the grain boundaries. In addition, slip bands and secondary cracks were observed around certain grain boundaries, and the slip band had a 45° angle along the grain boundary. The morphology indicated that grain boundary sliding occurred around the grain boundaries, as presented in Figure 6a,c. Further observation indicated that the amount of slip bands at crack boundaries was lower compared to the non-cracked grain boundaries near principal cracks. Generally, the crack type is related to gas phase embrittlement, due to the diffusion enrichment of oxygen at grain boundaries [29]. Meanwhile, the enrichment of oxygen leads to a weaker bonding force and results in grain boundary cracking within a short time [30]. From Figure 6b, we can see the grain boundaries with additional amount of precipitated η phase did not produce cracking although subjected to plastic deformation. The result suggests that the precipitated η phase at grain boundaries hindered propagation of secondary cracks.

**Figure 7:**
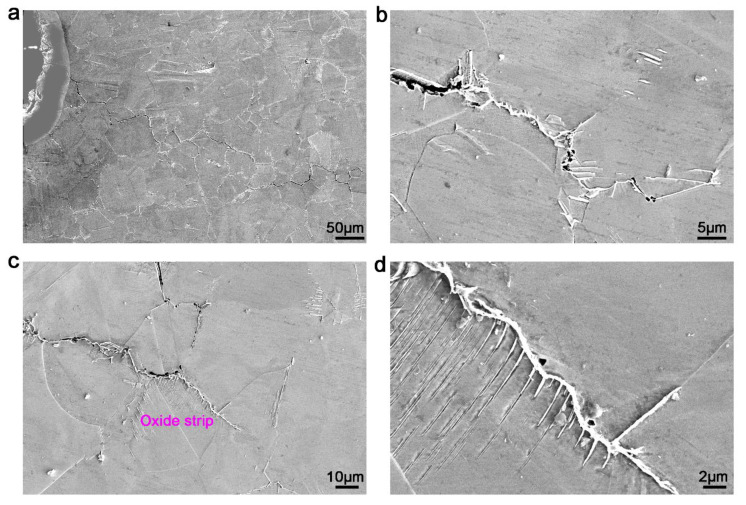
Propagation paths of cracks in 718Plus alloy withhold-time of 90 s. (**a**) Propagation path; (**b**) precipitation at grain boundaries; (**c**) morphology at crack tip; (**d**) oxide strip at crack tip.

Figure 7 presents the propagation paths of fatigue cracks in 718Plus alloy at the testing temperature of 704 °C and a loading mode of a 15 s-90 s-15 s-2 s trapezoidal waveform under air condition. Compared to the crack propagation path of the specimen at 5 s of holding time, no apparent difference existed between the two results. As presented in Figure 7a, with a 90 s hold-time at 704 °C, the fatigue cracks in the 718Plus alloy were initially generated and propagated along the grain boundaries at approximately 45° angle to the applied stress. These cracks propagated in a zigzag manner along a direction nearly perpendicular to the stress, while the expansion path also followed the grain boundaries (Figure 7b–d).

However, it could be noted that, a long oxide strip appeared near the crack tip at 90 s hold-time, which was longer than the oxide strip near the crack tip that with 5 s hold-time, as presented in Figure 7d. Moreover, there were no significant cracks caused by the connected voids between the oxide strip and the substrate. From theoretical analysis, it was inferred that the oxide strip formation caused the crack tip to close [10,12,31]. Thus, it caused the real crack tip to move backwards, consequently decreased the effective Δ*K*, which inevitably affected the crack-growth rate. Numerous slip bands appeared near the crack tip, while apparent oxide strips appeared on the slip bands close to one side of the grain boundaries. These changes indicated that oxidation preferentially occurred at these positions as channels for oxygen rapid diffusion, such as grain boundaries and slip bands. Through the energy spectrum analysis of the oxide strip at the crack tip, it was found that the oxides were mainly rich in Ni, Fe and Cr, along with low amounts of Nb, Mo and Al, as shown in Table 4.

## 4. Discussion

Historically, time-dependent (fatigue-crack growth) FCG was first attributed to fatigue–creep interaction. It was believed that creep can increase the FCG rate of alloys by creep damage, grain boundary cavity formation, linkage and creep crack growth [32]. With the increase of hold-time, the effects of creep and oxidation increase significantly, the creep damage at the grain boundaries is aggravated, and the oxide-induced embrittlement effect increases, that all promote the propagation of cracks [33]. Therefore, it can be concluded that the propagation rate of fatigue cracks in superalloys increases with the increase of hold-time. Many scholars have also confirmed this through experimental studies. For example, Li Xiao et al. [22] studied the propagation rate of fatigue cracks in FGH95 alloy at 650 °C with the hold-time of 90 s and 5 s under air environment. The experimental result showed that the crack-growth rate of specimen with a 90 s hold-time was approximately 5 times higher than that with a 5 s hold-time during the steady-state propagation stage. R. Jiang et al. [34] reported that additional oxidation effects on propagation caused by higher temperature or prolonging dwell time appear limited, whereas a prolonged dwell period seems to instead promote additional creep process, which further enhance crack growth, especially at higher temperature.

Conversely, the results in Figure 4 indicated that the longer the creep acting time was, the lower the fatigue-crack growth rate in 718Plus alloy was, i.e., the creep inhibited the fatigue-crack propagation of 718Plus alloy. This effect was more apparent within lower range of stress intensity factor Δ*K*. Moreover, the difference of fatigue-crack growth rates in 718Plus alloy between with a 90 s hold-time and with 5 s hold-time became lower with the increase of Δ*K*, indicating that the creep effect was not evident at high Δ*K*. The similar phenomenon was confirmed in Inconel 718 and Waspaloy alloys under specific experimental conditions [20,35]. The threshold for fatigue cracking in Inconel 718 alloy (Δ*K*_th_) did not decrease, but it gradually increased from 427 °C to 649 °C [35]. Further analysis confirmed that this effect was mainly related to the crack closure mechanism, which was caused by high-temperature oxidation, i.e., oxide-induced crack closure (OICC). In addition, the creep also suppressed propagation of fatigue cracks at 760 °C in Waspaloy alloy at a lower Δ*K* [20]. It was believed that the phenomena occurred due to the creep-induced stress relaxation and stress concentration at the crack tip. Only in the final stage of the test, creep damage led to cavity nucleation and growth at the grain boundaries, which accelerated the fatigue-crack growth rate of the alloy. Therefore, the beneficial effect of creep on fatigue-crack propagation of superalloy has been reported for only specific alloys, temperature and environment, and researchers have different opinions on the mechanism.

In this experiment, it could be deduced from the oxide morphology of the crack tip at different hold-times that the oxide strip length at the crack tip root at 90 s was significantly longer compared to the morphology at 5 s hold-time, because oxides formed more easily around the crack tip with a 90 s hold-time. However, the fatigue effect played a major role within 5 s hold-time, the crack tip demonstrated continuous opening and closing under the repeated action of stress. Subsequently, it was difficult to form the interconnected oxides. For the specimen within 90 s hold-time, the cracks remained in the open state for a long time, while the crack tip tended to form interconnected oxides during high-temperature thermal exposure. The formation of interconnected oxides led the crack tip to close and caused the backward movement of the crack tip. Thus, the effective stress field intensity factor decreased, and the fatigue-crack growth rate decreased.

At high value of Δ*K*, on one hand, the cracking wedge angle of the crack tip increased. On the other hand, the fatigue-crack propagation was accelerated, and step length interval of crack propagation was shortened. Consequently, it was more difficult to form a continuous stable oxide strip at the crack tip inside specimen with a 90 s hold-time, indicating that the effect of oxides on the propagation rate of the fatigue cracks gradually decreased. This was also the reason for the propagation rate difference when Δ*K* decreased, while the propagation rates were similar as Δ*K* increasing. Since the chemical compositions and strengthening mechanisms of different alloys are not the same, the resultant oxidation mechanisms and oxidation rates are accordingly different. Therefore, the effect of oxidation on the propagation rate of fatigue cracks in different alloys is different under the effect of high-temperature creep. The DFCGR reduced significantly in vacuum compared to air, which could be a result of an oxidation related phenomenon [36]. The stress relaxation caused by creep can relieve the stress concentration at the crack tip [20]. However, this viewpoint cannot explain the evident decrease in fatigue-crack growth rate at low oxygen partial pressure environment. In addition, the reduced effect of creep on propagation rate with increase in Δ*K* is also difficult to explain. Therefore, the OICC mechanism is a more probable mechanism for the propagation rate reduction of fatigue cracks in the 718Plus alloy under creep.

To date, it remains unclear why the beneficial effect of creep on fatigue-crack propagation of superalloy occurs only in specific alloys and at a high temperature. It may be attributed to three factors: alloy composition, microstructure and test condition. The oxidation sensitivity depends strongly on the alloy composition. For the three superalloys, Inconel 718, 718Plus and Waspaloy, it was found that they all have the beneficial creep effect and may relate to the similar oxidation mechanism at high temperature [8]. In addition, the three alloys all have grain boundary precipitations: M_23_C_6_ in Waspaloy alloy, δ phase in Inconel 718 alloy and η–Ni_3_Al_0.5_Nb_0.5_ in 718Plus alloy [8]. Grain boundary precipitations in the superalloys can inhibit the fatigue-crack propagation and facilitate the emergence of oxidation strip at grain boundaries. Finally, the test conditions, including temperature, loading process, environment, etc., also need to be conducive to the emergence of oxidation strip at grain boundaries because the emergence of oxidation strip at grain boundaries is the premise of OICC. On the other hand, the formed oxides could restrain the sliding of grain boundaries and prevent any apparent sliding near the cracks. Therefore, the beneficial effect of creep on fatigue-crack propagation in superalloys plays a role in specific alloys and test conditions.

The propagation paths of the crack tip in Figure 6c,d showed that the cracking first occurred at the grain boundary in front of the crack tip, rather than propagating forward along the crack tip. Moreover, the secondary cracks also propagated along grain boundaries (Figure 6a,d), indicating that grain boundaries were the main pathways of high-temperature fatigue cracks and weak regions affecting the propagation rate of fatigue cracks. This was because under the action of stress, the maximum stress position was not at the crack tip, but in front of the crack tip, which was related to the Δ*K* value, as seen in Figure 6. Grain boundaries owning a weaker bonding force (such as the grain boundaries with lower η phase amount) and a certain angle with the principal stress will slide first, subsequently lead to cracking. The microcracks would connect to the crack tip, producing step-wise crack propagation. In addition, as presented in Figure 6d, there were many voids in the interface between the oxides and grain boundaries. These voids expanded and connected to each other leading to the generation of new cracks. The above factors can promote the continuous formation of secondary grain boundary cracks.

## 5. Conclusions

In this study, the dwell-fatigue-crack growth rates of 718Plus alloy at two creep times were evaluated. The test results confirmed that creep had an abnormal inhibitory effect on the propagation of fatigue cracks in 718Plus alloy at high temperature of 704 °C, i.e., the longer the creep time, the lower the propagation rate of fatigue cracks. In particular, under the lower Δ*K* condition, the fatigue-crack growth rate with a 90 s hold-time was one order of magnitude lower than that with 5 s hold-time. In contrast, as Δ*K* increased, the difference between the two rates gradually decreased, and the creep effect became less apparent.

Based on the combined analysis of microstructure, high-temperature oxidation rate, propagation path of cracks and the step interval of 718Plus alloy under different conditions, it was confirmed that the effect of creep on lowering the fatigue-crack growth rate was related to OICC mechanism, due to the oxidation at lower Δ*K*. These findings can provide an experimental and theoretical basis for further research on improving the fatigue-crack propagation ability of 718Plus alloy and its engineering application.

## Figures and Tables

**Table 1:** Chemical composition of the 718Plus alloy used in this study (in wt%).

C	Cr	Mo	Nb	Ti	Al	Co	Fe	W	Ni
0.044	18.98	2.78	5.45	0.75	1.51	8.99	9.28	1.07	Bal.

**Table 2:** Mechanical properties at 704 °C of experimental superalloy.

Specimen	Tensile Strength (MPa)	Yield Strength (MPa)	Elongation (%)	Reduction of Cross-Section Area (%)
718Plus	1219	1051	23	28

**Table 3:** Parameters corresponding to the crack-growth rates with 5 s and 90 s hold-times, respectively.

Hold-Time	5 s	90 s
Transition from crack initiation to propagation (mm)	0.1	0.2
Transition from crack propagation to rupture (mm)	2.4	1.6
Cycle number of crack initiation (*N*_q_)	1215	2209
Starting cycle numbers of crack propagation (*N*_n_)	1327	5401
Starting cycle numbers of crack rupture (*N*_s_)	1463	6460
Cycle number of fracture (*N*_f_)	1505	6477
Proportion of crack initiation (P*)	0.88	0.83
Proportion of steady-state propagation (*P*)	0.09	0.16

**Table 4:** Oxide analysis at crack tip in 718Plus alloy.

Elements	O	Al	Ti	Cr	Fe	Ni	Nb	Mo
wt%	34.83	1.3	0.51	13.67	13.86	30.31	3.9	1.63
at%	65.53	1.45	0.32	7.92	7.47	15.54	1.26	0.51
